# The use of ultrasound to identify milk ejection in women – tips and pitfalls

**DOI:** 10.1186/1746-4358-4-5

**Published:** 2009-06-01

**Authors:** Donna T Geddes

**Affiliations:** 1 M310, Biomedical, Biomolecular and Chemical Sciences, Faculty of Life and Physical Sciences, The University of Western Australia, Western Australia, Australia

## Abstract

Diagnostic ultrasound imaging of the breast has been limited principally to the abnormal, non-lactating breast. Due to the rapid improvement of imaging technology, high-resolution ultrasound images can now be obtained of the lactating breast. Ultrasound scanning techniques, however, require modifications to accommodate the breast changes that occur in lactation. Furthermore, the function of the breast with regard to milk ejection can be assessed with ultrasound by identification of milk duct dilation and milk flow. At milk ejection, the echogenic duct walls expand as milk flows forward towards the nipple. Milk flow appears as echogenic foci rapidly moving within the milk duct. This paper provides a detailed description of the ultrasound technique used for the detection and reviews nuances associated with the procedure.

## Introduction

Although milk ejection is integral to lactation and thus the survival of the species, the lack of knowledge regarding milk ejection in women, in comparison to other lactating and dairy animals, is surprising. Oxytocin is a vital hormone for the maintenance of lactation, yet there are no studies to determine if women with lactation problems, such as low milk production, have normal milk ejections/oxytocin release. Since both milk synthesis and milk ejection must occur to ensure successful lactation, methods are required to assess both processes. Currently, milk production can be estimated in a relatively non-invasive way by the test weigh method [1], whereas milk ejection can be assessed using ultrasound imaging [2]. Recent improvements in ultrasound equipment have allowed high resolution imaging of the lactating breast [3]. Ultrasound monitoring offers a safe, non-invasive alternative to both serial blood sampling (to detect oxytocin levels) and intra-ductal pressure measurements (cannulation of a duct through the nipple pore). Furthermore, ultrasound duct dilation has been correlated with milk flow rates during pumping [4,5]. This paper will describe the ultrasound technique and its analysis in detail and also provide visual examples of milk ejection to aid the clinician or researcher to apply this method.

### Milk ejection

**Figure 1:**
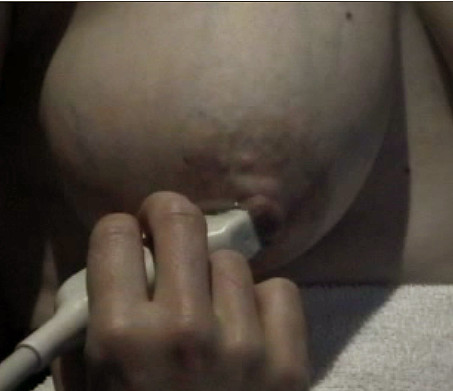
**Photograph of the right areola of a lactating woman prior to milk ejection**. The milk ducts directly superior to the nipple are very superficial and can be seen as bulging under the skin.

**Figure 2:**
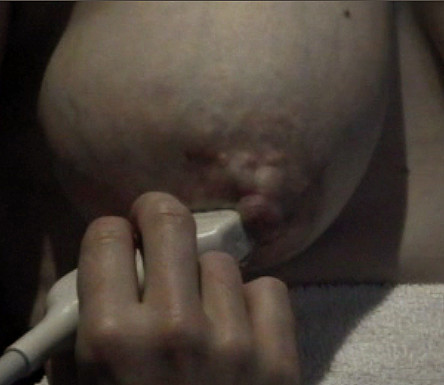
**Photograph of the right areola of a lactating woman at milk ejection**. Note the increased swelling of the areola. This is due to the superficial ducts expanding at milk ejection.

Lactocytes (secretory mammary epithelial cells) line the alveoli of the lactating breast and synthesize milk. In women, the greater portion of the milk is stored in the alveolar region until required by the suckling baby. Milk ejection is the process by which milk is forced into the larger ducts to become available for removal by either the infant or breast pump. Stimulation of the nipple causes the release of oxytocin from the posterior pituitary into the bloodstream. Oxytocin then binds to receptors on the myoepithelial cells that surround the milk-filled alveoli, causing them to contract and thereby forcing the milk into the milk ducts [6,7]. Milk ejection is a transient phenomenon lasting between 45 seconds and 3.5 minutes [8-10]. Oxytocin is therefore released in a pulsatile fashion with multiple ejections usually occurring during either a breastfeed or pumping session [9,10]. Milk ejection is critical for successful breastfeeding and continued milk synthesis [11], as little milk (approximately 2.7 mL; range 0 to 10.3 mL) can be removed prior to milk ejection [12]. Typical maternal sensations of milk ejection include tingling, pins and needles, pain or pressure in the breast and milk flow from the breast. Occasionally, the mother may experience systemic symptoms such as nausea, warmth or thirst [6,13]. These sensations are often strongest for the first milk ejection, waning for subsequent ejections during the breastfeed/pump. In the absence of sensations of milk ejection during breastfeeding the infant may change its sucking behaviour to a regular, more rhythmic pattern, whereas during pumping, milk jets may be observed. In addition, we have noticed that the areola region becomes more full and tense and this appears to be more marked in mothers with larger (> 4 mm) superficial ducts (Figures 1 and 2; Additional file 1). Methods for the detection of milk ejection have been developed, such as frequent sampling of maternal blood to detect oxytocin [9] and measurement of intra-ductal pressure by the cannulation of a milk duct through a nipple pore [14]. Detection of an increase in pressure is associated with the release of oxytocin and milk ejection. However both of these procedures are invasive and stressful. It is possible that the stress associated with the procedure itself may impair milk ejection by inhibiting the release of oxytocin, resulting in decreased milk yield [15]. In addition, measurement of intra-ductal pressure carries the added risk of the introduction of infection to the breast. Alternatively, ultrasound is a convenient, cost-effective method of confirming milk ejection during either breastfeeding or breast pumping [4], particularly if the infant/pump is removing very small quantities of milk.

### Ultrasound equipment

#### Technical requirements

Ultrasound imaging of the lactating breast requires equipment capable of resolving the ductal structures of the breast. The diameter of the main milk ducts (beneath the areola) in the lactating breast range from less than 1 millimetre up to 10 millimetres [2,16]. The near field resolution of the ultrasound system (subcutaneous portion of the breast) should be as high as possible, as the ducts are very superficial in the areola region. In some mothers there is no visible intervening tissue between the duct and the overlying skin. Depending on the resolution of the system, a standoff may be necessary to improve focusing of the transducer superficially. An electronically focused linear array transducer with a frequency of 7–12 MHz and multiple focal zones is appropriate for breast imaging and, in particular, for the identification of milk ejection [17].

#### Ultrasound settings

The time compensation curve (compensates for the normal attenuation of the sound waves in the tissue) is generally a gentle slope. The gain setting compensates for attenuation of the ultrasound beam without discriminating for depth, thus amplifying all of the returning echoes [18]. Too high a gain setting will eliminate visualisation of the ductal walls, whereas too low a gain setting may eliminate the visualisation of milk flow at milk ejection. A compromise may be necessary in some women, particularly those where duct dilation is minimal and one must rely more on milk flow for identification of milk ejection. One focal zone focused at the level of the monitored duct is normally sufficient, however two focal zones may be considered in women with larger ducts (>5 mm). The depth setting should be optimized to show the main ducts that are superficial in the breast (20 mm). Too great a depth setting will result in the ducts appearing smaller and increase the possibility of not detecting a very small increase in duct diameter at milk ejection. The power setting should be high enough to ensure adequate visualisation, but can often be reduced due to the limited depth of insonation. The dynamic range is approximately 60 to 70 dB, depending on the particular machine.

### Ultrasound technique for detection of milk ejection in the lactating mother

**Figure 3:**
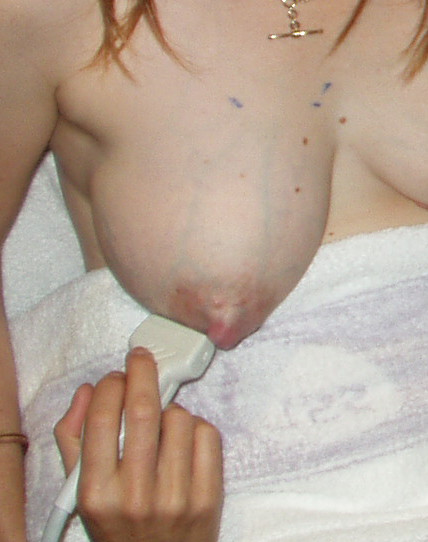
**Ultrasound scanning position for detection of milk ejection in the lactating breast**. The breast that is not suckled/expressed is monitored using a high frequency linear array ultrasound transducer. The milk duct monitored is in the lateral portion of the breast near the base of the nipple. Minimal pressure must be used to avoid compression of the duct.

The mother should be seated comfortably during a scan to facilitate either breastfeeding or pumping in a natural position. Prior to either breastfeeding/pumping a milk duct in the un-suckled/non-expressed breast is identified and monitored for the session. The mother should be instructed to limit her movements where possible in order to reduce movement artefact during the scan. Milk ducts conducive to monitoring tend to be ducts greater than 1 mm in diameter beneath the areola in the lateral portion of the breast (Figure 3). The probe is rotated until the long axis of the portion of the duct to be monitored is obtained. The gain is set so that the duct is not completely anechoic (devoid of echoes, black) and milk flow can then be identified. It is prudent to use colour Doppler flow imaging [19] to discriminate between milk ducts and blood vessels, particularly when the milk ducts are very small. It is critical that the ultrasound technician adopts a comfortable, ergonomically correct position, as the transducer must be held still for the duration of the breastfeeding/pumping session. Consistently light pressure also must be applied to ensure that the milk duct is not compressed thereby reducing duct dilation at milk ejection. Testing compression levels prior to the scan is wise, to ensure the duct is not already partially compressed prior to commencement of the monitoring period. The scan begins as soon as either the baby attaches to the mother's breast or the breast pump is switched on. A marker can be used to indicate the beginning of the feed/pump. It is also useful to instruct the mother to indicate if she senses milk ejection and to mark this on the scan for later analysis. Identification of milk ejection enables one to switch the breast pump to an expression pattern if this is an available feature. In addition, the collection bottle can be changed to determine how much milk is removed prior to milk ejection [20]. An accurate measurement of the milk removed before milk ejection can be made by weighing the collection bottle with accurate digital scales. The difference in weight between the bottle containing the milk removed prior to milk ejection and the empty bottle is the amount of milk removed prior to milk ejection. The difference in grams is equivalent to millilitres; for example one gram of breast milk corresponds to approximately one millilitre of breast milk. It is essential that the scan be videotaped to allow careful retrospective analysis, particularly in difficult cases.

### Normal ultrasonic appearances

#### Ultrasonic appearances of the lactating breast

**Figure 4:**
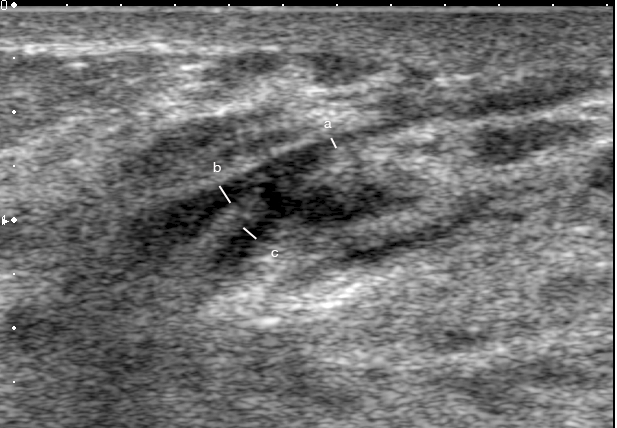
**Ultrasound image of the main milk duct in a lactating woman**. The milk ducts are displayed on ultrasound as hypoechoic (black) branching structures. The measured main milk duct is barely imperceptible with a diameter of 0.4 mm (a). The merging milk ducts measured 0.8 mm (b) and 0.75 mm (c).

**Figure 5:**
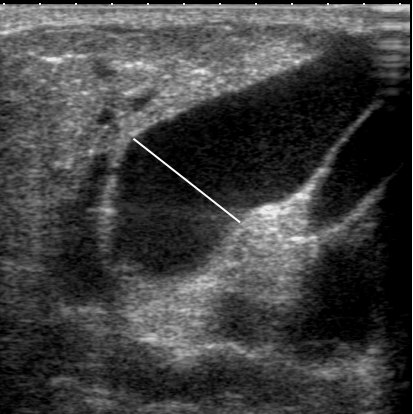
**Ultrasound image of the main milk duct in a lactating woman**. The main milk duct is displayed on ultrasound as a hypoechoic (black) structure with echogenic walls (white). This main milk duct is very large, measuring 9.2 mm in diameter.

Many of the structures of the lactating breast have a similar appearance to that of the non-lactating breast. Particular features of the lactating breast that should be considered are that the ductal structures are generally small, approximately 2–3 millimetres in diameter, and easily compressible [21]. However, ducts may be less than 1 millimetre in diameter (Figure 4) and as large as 10 millimetres in diameter (Figure 5). Furthermore, the internal lumen of ductal structures are not completely anechoic and contain small echogenic foci that most likely represent fat globules in the milk [21]. The fat content of the milk near the nipple is higher in a drained breast compared to a full breast, accounting for the variability of the echogenicity of the milk within the ducts [1] (Table 1). More detailed descriptions regarding the anatomical ultrasonic appearances of the lactating breast can be found in Geddes [3].

**Table 1:** The ultrasonic appearances of the structures of the lactating breast (adapted from Geddes [3])

**Structures of the breast**	**Ultrasonic appearance of the lactating breast**
Milk ducts	Hypoechoic, can contain echogenic flecks representing milk fat globules Echogenic walls may be visible when insonated at 90 degrees Easily compressible Distend at milk ejection Resting state 2 mm (range;1–10 mm)
Skin	Hyperechoic Increased thickness in the areola region
Coopers ligaments	Hyperechoic
Stromal fibrous tissue	Predominantly hyperechoic – tends to be more echogenic with more milk in the breast
Adipose tissue	Hypoechoic, variable amounts Large breasts often contain a large proportion of adipose tissue
Arteries and veins	Hypoechoic, demonstrate blood flow on colour Doppler imaging

#### Ultrasound appearances of milk ejection

**Figure 6:**
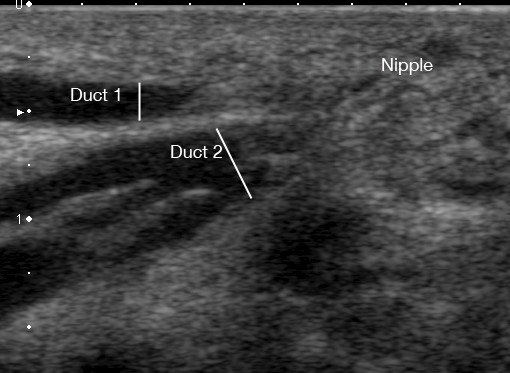
**Ultrasound image of a milk ducts in the human lactating breast prior to milk ejection**. Two main milk ducts are displayed on ultrasound as a hypoechoic (black) structure with echogenic walls (white). Duct 1 is more superficial (1.95 mm) than Duct 2 (3.72 mm). Duct 2 has three merging branches.

**Figure 7:**
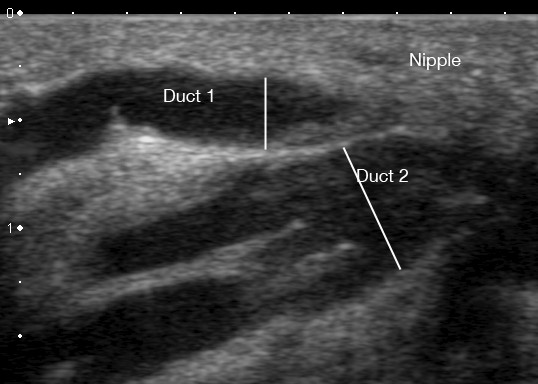
**Ultrasound image of a milk ducts in the human lactating breast at milk ejection**. Two main milk ducts are displayed on ultrasound as a hypoechoic (black) structure with echogenic walls (white). Duct 1 is more superficial and has increased from 1.95 mm to 3.44 mm in diameter. Duct 2 has increased from 3.72 mm to 6.24 mm.

**Figure 8:**
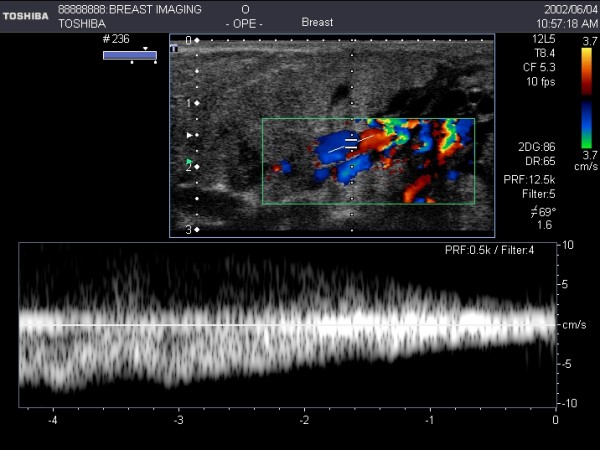
**Colour flow Doppler imaging of milk flow within multiple ducts at milk ejection**. Colour Doppler detected the movement of milk within the milk duct at milk ejection. Using pulsed wave Doppler the velocity of milk flow can be measured. Measurement of milk flow within a milk duct is difficult due to Doppler angle, the transient nature of milk ejection and low velocity milk flow.

The ultrasound appearances of milk ejection are summarized in Table 2. At milk ejection, duct dilation is observed (Figure 6 and 7) due to increased intra-ductal pressure and the forward flow of milk can also be identified as the echogenic flecks (fat globules in the milk) moving towards the nipple (Additional file 2). There is variability in the degree of duct dilation between women, with some having large increases in duct diameter (Additional file 3) and others having minimal increases (Additional file 2). Milk flow is difficult to detect with colour Doppler flow imaging, but is possible at very low flow settings (Figure 8). During the second half of a milk ejection, reverse flow of the milk is often observed as the duct reduces back to its resting diameter (Additional file 4) [2]. Multiple ejections are shown as pulsatile increases and decreases in duct diameter. An average of 2.5 milk ejections have been detected during a breastfeed (range 0 to 9) [2] and 4 to 5 milk ejections detected for a fifteen minute expression with an electric breast pump (range 1 to 12) [4].

**Table 2:** Summary of ultrasonic features particular to milk ejection in the lactating breast

**Milk ejection**	**Ultrasonic change at milk ejection**	**Clinical signs**
First half*	Milk duct diameter increases Milk flow (echogenic flecks) towards the nipple	Sensation of milk ejection felt Pumping – visualisation of jets of milk, rapid increase in milk flow Breastfeeding – change in sucking to slower more rhythmical pattern If flow is very fast the infant may pull off the breast
Second half**	Milk duct diameter decreases Milk flow reverses back into the breast	Sensation of milk ejection Pumping – visualisation of milk jets, slowing of milk flow Breastfeeding – slow, more rhythmical pattern Infant may discontinue feeding during milk ejection if appetite met

*First half of milk ejection is defined as the initiation of duct dilation until peak duct diameter is reached.

**Second half of milk ejection is the decrease in duct diameter from peak diameter to either baseline diameter or the beginning of another duct dilation.

#### Analysis of ultrasound for milk ejection

**Figure 9:**
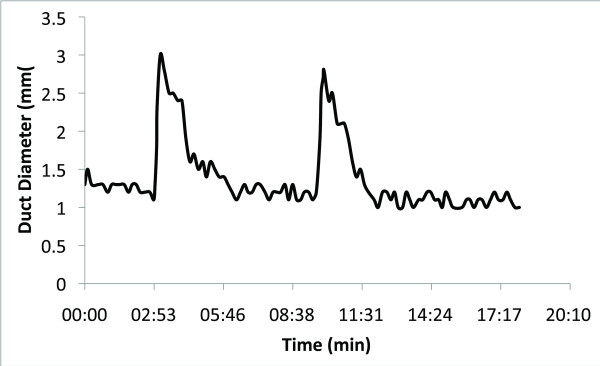
**Plot of duct diameter measured using ultrasound imaging during a 15-minute expression session using an electric breast pump**. Duct diameter increased rapidly at 2 minutes and 50 seconds and 9 minutes 15 seconds. Both duct dilations (milk ejections) lasted approximately 180 seconds before duct diameter returned to the initial diameter.

**Figure 10:**
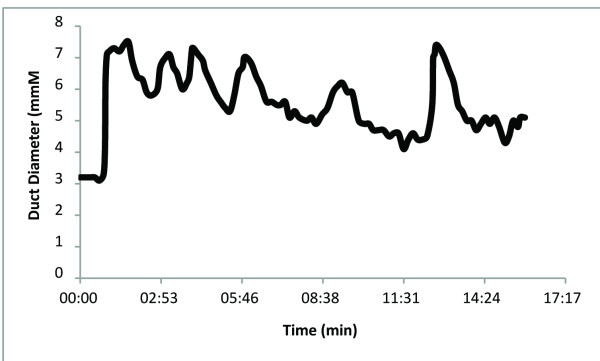
**Plot of duct diameter measured using ultrasound imaging during a 15-minute expression session using an electric breast pump**. Six duct diameter dilations (milk ejections) lasting approximately 100 seconds each were detected during this pumping session.

Retrospective analysis of the videotape is carried out in order to measure milk duct diameter every three to twenty seconds, that is, at times when the breast has stabilized from movement of the mother, baby or the positioning of the transducer. This enables one to plot milk duct diameter over the length of the feed/pumping session. These plots enable better evaluation of both the number and the duration of the milk ejections. Figures 9 and 10 show the variation of milk ejection patterns between women. The duration of milk ejection has been estimated from the beginning of an increase in duct diameter to the beginning of the next increase in duct diameter [4].

### Clinical relevance of monitoring for milk ejection

The milk ejection process is critical to successful lactation, yet there is currently a lack of methods to assess whether or not milk ejection has occurred. This is particularly relevant for the proportion of women who do not sense milk ejection. Milk flow rate during pumping has been associated with milk ejection imaged as an increase in duct diameter, therefore measurement of flow rate may be useful to confirm milk ejection in women who are pump dependent, such as mothers of preterm infants and those women who can pump successfully. Unfortunately, a proportion of women are unable to express substantial amounts of milk with a breast pump, and for these women ultrasound imaging would provide a means of verifying a normal milk ejection reflex.

It is important that ultrasound technicians performing a diagnostic breast scan be familiar with both the ultrasonic and clinical signs of milk ejection. A woman who volunteered for a research study had a history of a prior ruptured breast abscess. As a result she had a soft lump that increased transiently in size during feeding. Scanning of the lump during breastfeeding showed that the lump contained functional milk ducts that expanded at milk ejection (Additional file 5) and was not a fluid filled cavity requiring aspiration. Spontaneous milk ejection may occur during scanning, particularly if either the breast is full of milk or the transducer stimulates the nipple initiating an oxytocin release. Furthermore, awareness of the symptoms of milk ejection may be helpful when scanning women who experience pain in their breasts. This would facilitate efforts to determine if the sensation of pain coincides with milk ejection.

## Conclusion

Ultrasound imaging is a reliable, non-invasive means of identifying duct dilation that occurs during milk ejection during either breastfeeding or breast expression. The examination requires high-resolution ultrasound equipment and specific attention to anatomical appearances specific to the lactating breast. This technique has many potential applications for both the research and clinical lactation field.

## Competing interests

The author receives a salary as part of a research grant provided by Medela AG.

## Supplementary Material

Additional file 1 **Milk ejection movie 1**. Outward signs of milk ejection may be observed as increasing bulging under the areola. This phenomenon is due to milk duct dilation and the superficiality of the ducts in this region. Milk can also be seen to drip from the nipple in some women at milk ejection. Click here for file

Additional file 2 **Milk ejection movie 2**. Ultrasound video of milk ejection in the un-suckled breast during breast pumping. Milk flow can be seen as movement of echogenic flecks within the milk duct at milk ejection. This woman has minimal duct dilation with the duct increasing from 2.0 mm to 2.6 mm. Note all of the ducts expand and display milk flow at milk ejection. Click here for file

Additional file 3 **Milk ejection movie 3**. Ultrasound video of milk ejection in the un-suckled breast during breast pumping. Milk flow can be seen as movement of echogenic flecks within the milk duct at milk ejection and duct dilation is obvious. Click here for file

Additional file 4 **Milk ejection movie 4**. Ultrasound video of milk ejection in the un-suckled breast during breast pumping. Forward milk flow (to the left of the image) is observed within the ducts initially and then backward flow (to the right of the image) occurs soon after when milk is not removed from the breast. Click here for file

Additional file 5 **Milk ejection movie 5**. Ultrasound imaging of a superficial palpable lump formed after a ruptured abscess in a previous lactation. The mass increases in size and the hypoechoic ductal structures within the mass also expand. Click here for file
